# Weak acids induce PGE_2_ production in human oesophageal cells: novel mechanisms underlying GERD symptoms

**DOI:** 10.1038/s41598-020-77495-z

**Published:** 2020-11-27

**Authors:** Daichi Sadatomi, Toru Kono, Sachiko Mogami, Naoki Fujitsuka

**Affiliations:** 1 Tsumura Kampo Research Laboratories, Tsumura & Co., Ibaraki, Japan; 2 Institute of Biomedical Research, Sapporo Higashi Tokushukai Hospital, Hokkaido, Japan

**Keywords:** Oesophageal diseases, Gastro-oesophageal reflux disease

## Abstract

The role of weak acids with pH values in the range of 4–7 has been implicated in the symptoms of gastroesophageal reflux disease (GERD). Prostaglandin E_2_ (PGE_2_) is associated with heartburn symptom in GERD patients; however, the precise productive mechanisms remain unclear. In this study, we revealed that exposure to weak acids increases PGE_2_ production with a peak at pH 4–5, slightly in human normal oesophageal cells (Het-1A), and robustly in oesophageal squamous carcinoma cells (KYSE-270). Release of PGE_2_ from the oesophageal mucosa was augmented by weak acid treatment in rat. Chenodeoxycholic acid (CDCA), a bile acid, upregulated cyclooxygenase-2 (COX-2) expression in Het-1A and KYSE-270 and induced PGE_2_ production in KYSE-270 cells. Weak acid-induced PGE_2_ production was significantly inhibited by cytosolic phospholipase A2 (cPLA2), ERK, and transient receptor potential cation channel subfamily V member 4 (TRPV4), a pH-sensing ion channel, inhibitors. Hangeshashinto, a potent inhibitor of COX-2, strongly decreased weak acid- and CDCA-induced PGE_2_ levels in KYSE-270. These results indicated that weak acids induce PGE_2_ production via TRPV4/ERK/cPLA2 in oesophageal epithelial cells, suggesting a role in GERD symptoms like heartburn. Interventions targeting pH values up to 5 may be necessary for the treatment of GERD.

## Introduction

Gastroesophageal reflux disease (GERD) is an inflammatory disease of the upper gastrointestinal tract characterised by heartburn and acid regurgitation. Damaged oesophageal mucosa caused by acid reflux may develop into Barrett’s oesophagus, which is a major risk factor for oesophageal adenocarcinoma^1,2^. Since the extent of mucosal damage is proportional to the acid reflux time in patients with GERD^3–6^, reflux with pH values < 4 is defined as “acid reflux” and is used as an index for the diagnosis and treatment of GERD. Advanced techniques for the measurement of oesophageal pH have shown that reflux events with values other than pH < 4, such as weak acids (i.e., pH 4–7), are possible responsible substances related to various GERD symptoms^7^. Several studies have reported that weak acids contribute to the pathogenesis of GERD symptoms in patients with proton pump inhibitor (PPI)-refractory GERD, which are considered problematic among the majority of clinicians^8–12^. Despite recent evidence for the role of weak acid reflux, the degree of risk and mechanisms by which it leads to GERD-related symptoms remain unclear.

Prostaglandin E_2_ (PGE_2_), an inflammatory mediator, is involved in various inflammatory diseases. Cyclooxygenase 2 (COX-2), a rate-limiting enzyme in PGE_2_ production, is overexpressed in dysplastic lesions of the oesophagus. In GERD patients, nonsteroidal anti-inflammatory drugs effectively inhibit heartburn symptoms^13,14^. Indeed, increased PGE_2_ production by acid exposure has previously been reported in healthy volunteers^15^. However, although bile induces PGE_2_ production in oesophageal epithelial cells by enhancing COX-2 expression^16–18^, few studies have examined the mechanisms of acid-stimulated PGE_2_ production. Especially, detailed investigations of the relationship between pH and PGE_2_ production in oesophageal epithelial cells are lacking.

In this study, we used several types of oesophageal epithelial cells and an animal model to investigate extracellular pH-dependent PGE_2_ production and the underlying mechanisms, with a focus on weak acids in comparison with bile acid stimulation. We also examined the effect of a Kampo medicine, hangeshashinto (HST), which reportedly alleviates reflux-related symptoms in patients with PPI-refractory GERD^19^, on PGE_2_ production.

## Results

### PGE_2_ production in oesophageal epithelial cells induced by acidic conditions and bile acid

**Figure 1:**
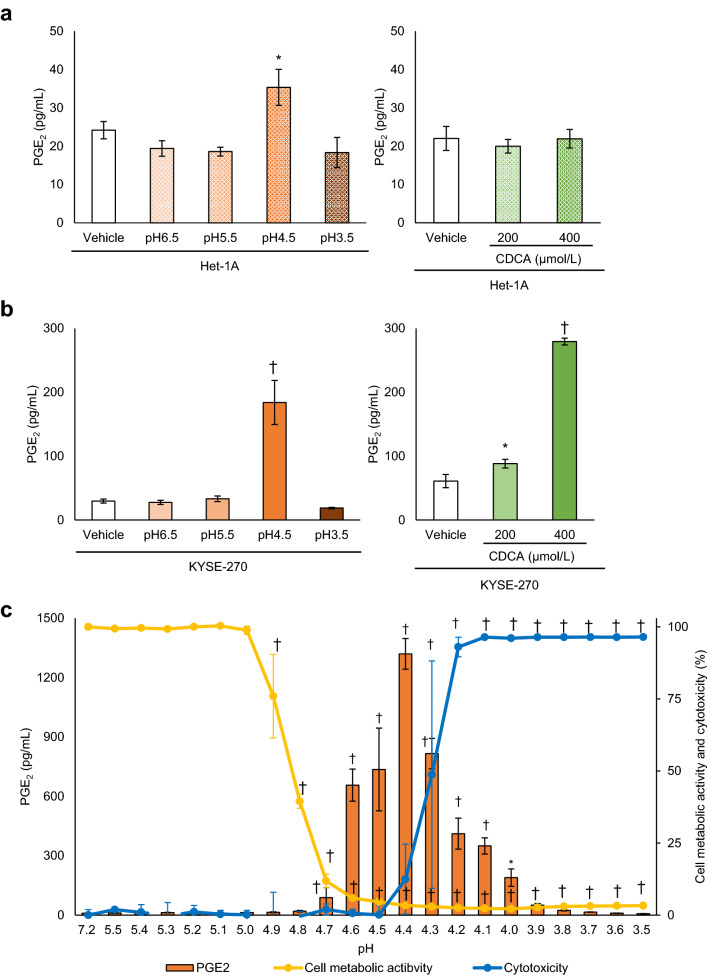
Prostaglandin E_2_ (PGE_2_) production in oesophageal squamous cell carcinoma KYSE-270 cells and normal oesophageal epithelial Het-1A cells. (**a**) A slight increase in PGE_2_ production was observed in oesophageal epithelial Het-1A cells cultured in fresh medium (pH 7.2) for 6 h after treatment with pH 4.5 medium (n = 3) for 2 h but not with media with other pH values or Chenodeoxycholic acid (CDCA; 200 or 400 μmol/L) (n = 4). (**b**) In oesophageal epithelial cell carcinoma KYSE-270 cells, PGE_2_ production was significantly increased by culture in fresh medium with pH 7.2 for 6 h after treatment with pH 4.5 medium for 2 h and with CDCA (200 or 400 μmol/L) (n = 3). (**c**) In KYSE-270 cells, an increase in PGE_2_ production was observed in medium with pH values from 4 to 4.7, with an increase in cytotoxicity at pH < 4.4. Cell metabolic activity was decreased at pH < 4.9 (n = 3). Data are presented as means ± SD. Statistical significance was determined by Dunnett’s test; *P < .01, ^†^*P* < .001, compared with vehicle (**a**,**b**) or pH 7.2 medium (**c**).

Human normal oesophageal epithelial cells, Het-1A cells, exhibited a slight increase in PGE_2_ production in response to pH 4.5 medium (*P* = 0.0031) but not acidic medium with other pH values or CDCA (200 or 400 μmol/L) (Fig. 1a). In the human oesophageal squamous cell carcinoma cell line KYSE-270, PGE_2_ production increased significantly after exposure to pH 4.5 (*P* = 0.00000014) and CDCA (CDCA [200 μmol/L]; *P* = 0.0090, CDCA [400 μmol/L]; *P* = 0.00000025) (Fig. 1b). PGE_2_ production increased with acidification as pH gradually decreased from 4.7 to pH 4.4 (Fig. 1c). Cell metabolic activity decreased in acidic medium at pH < 4.9, and cytotoxicity increased at pH values < 4.4 (Fig. 1c). In oesophageal adenocarcinoma cells, we observed significant but slight increase in PGE_2_ production by stimulation at pH 4.25–4.75 in KYAE-1 cells^20^, however no response was shown in FLO-1 cells (Supplementary Fig. S1).

### Weak acid treatment induces PGE_2_ production from rat oesophageal mucosa in vivo

**Figure 2:**
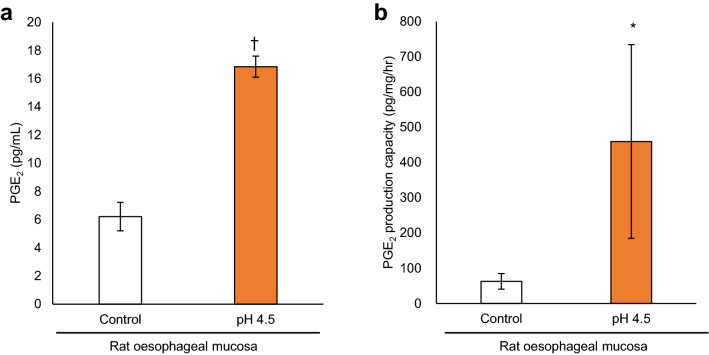
Weak acid induces PGE_2_ production in rat oesophageal mucosa. (**a**) PGE_2_ levels in the perfusate were increased by perfusing pH 4.5 solution to rat oesophagus at a rate of 500 μL/min for 1 h. (**b**) In oesophageal mucosa collected at 1 h after perfusion of a pH 4.5 solution, PGE_2_ production capacity significantly increased compared to that in the control group. Data are presented as means ± SD (n = 5). *PGE*_*2*_ prostaglandin E_2_. Statistical significance was determined by Student’s or Aspin-Welch’s t-test; *P < .05, ^†^*P* < .001.

Experiments were conducted using an animal model to examine whether weak acid also induces PGE_2_ production in vivo; we found that perfusion of a weak acid into the oesophageal lumen significantly increased PGE_2_ release in the perfusate of rats (Fig. 2a). After exposure to a weak acid, the oesophageal mucosa exhibited a marked increase in PGE_2_ production capacity compared with that in the PBS-treated group (Fig. 2b).

### Involvement of cyclooxygenases in PGE_2_ production in oesophageal epithelial cells

**Figure 3:**
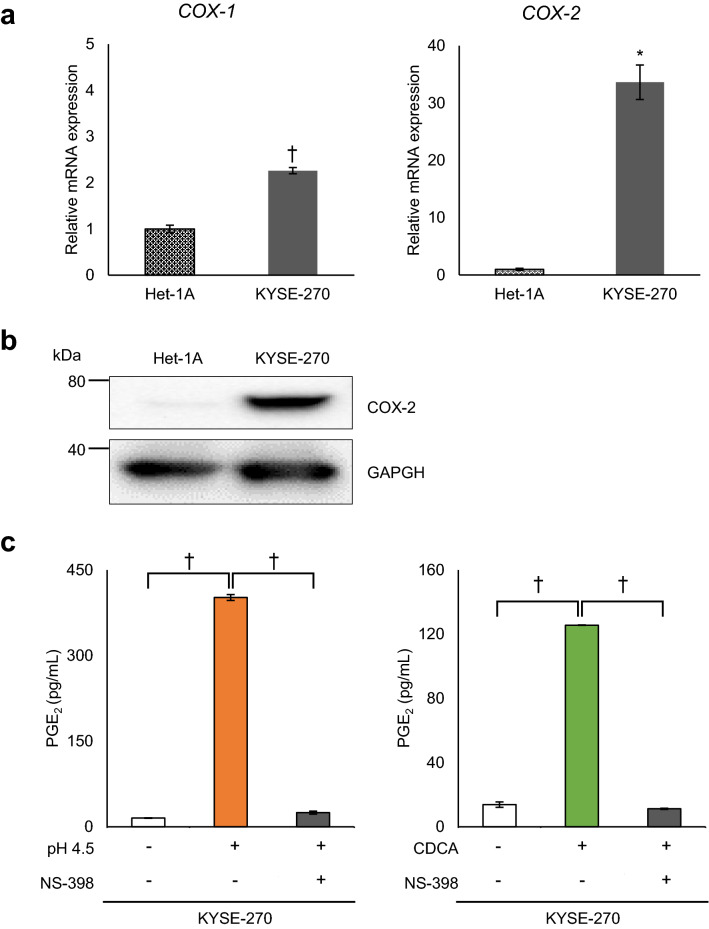
Basal expression levels of COX-1 and COX-2 in Het-1A and KYSE-270 cells. (**a**) The basal gene expression levels of cyclooxygenase-1 (*COX-1*) and cyclooxygenase-2 (*COX-2*) in KYSE-270 cells were higher than those in Het-1A cells. (**b**) The basal level of COX-2 protein expression was substantially higher in KYSE-270 cells than in Het-1A cells. The shown blots were cropped to improve the conciseness and the full-length blots are presented in Supplementary Fig. S6. (**c**) Treatment with the COX-2-selective inhibitor NS-398 (0.2 μmol/L) significantly inhibited PGE_2_ production in KYSE-270 cells treated with pH 4.5 medium or with 400 µmol/L CDCA. Data are presented as means ± SD (n = 3). *PGE*_*2*_ prostaglandin E_2_; *CDCA* chenodeoxycholic acid. Statistical significance was determined by Student’s or Aspin–Welch's *t*-test or Tukey–Kramer test; *P < .01, ^†^*P* < .001.

KYSE-270 was used to investigate the mechanism by which PGE_2_ is produced by “weak acid stimulation” and pH 4.5 since there was a sufficient PGE_2_ production without prominent cell death. *COX-2* mRNA levels in KYSE-270 cells were markedly higher than those in Het-1A cells, whereas cyclooxygenase-1 (*COX-1*) expression was slightly higher in KYSE-270 than in Het-1A (Fig. 3a). In a western blot analysis, COX-2 protein expression levels in KYSE-270 cells were also markedly higher than those in Het-1A cells (Fig. 3b). In KYSE-270 cells, the induction of PGE_2_ production by pH 4.5 medium and CDCA was inhibited by the selective COX-2 inhibitor NS-398 (Fig. 3c).

### Distinct mechanisms by which pH 4.5 and CDCA induce PGE_2_ production

**Figure 4:**
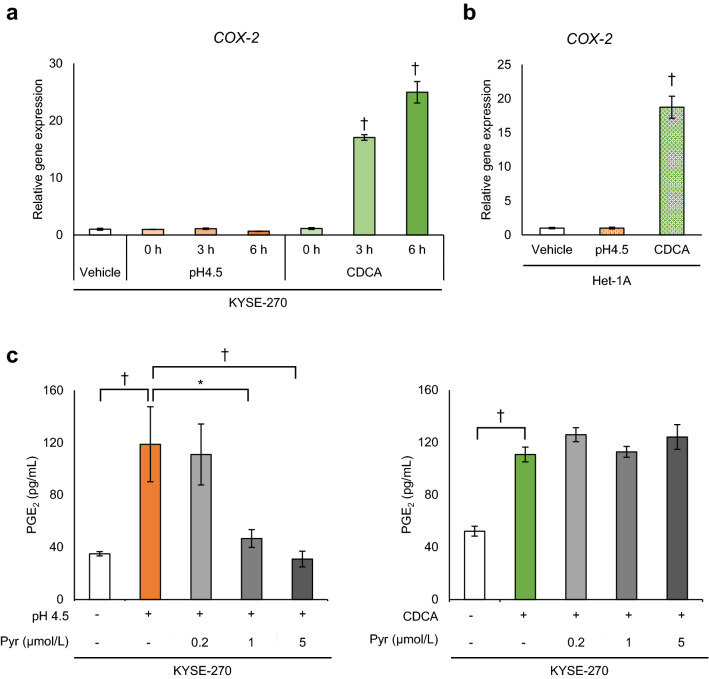
Different mechanisms underlying PGE_2_ production induced by pH 4.5 and CDCA. (**a**) *COX-2* expression increased significantly in KYSE-270 cells cultured in fresh medium with pH 7.2 for the indicated time after treatment with CDCA (400 μmol/L) for 2 h but not with pH 4.5 medium. (**b**) *COX-2* expression increased in fresh medium (pH 7.2) at 6 h after treatment with 400 μmol/L CDCA but not with pH 4.5 medium for 2 h in Het-1A cells. (**c**) Treatment with a cytosolic phospholipase A2 (cPLA2) inhibitor Pyrrophenone (Pyr; 0.2, 1, and 5 μmol/L) suppressed PGE_2_ production induced by pH 4.5 but not by CDCA (400 μmol/L) in KYSE-270 cells. PGE_2_, prostaglandin E_2_; CDCA, chenodeoxycholic acid; COX-2, cyclooxygenase-2. Data are presented as means ± SD (n = 3). Statistical significance was determined by Dunnett’s test or Tukey–Kramer test; *P < .01, ^†^*P* < .001.

In KYSE-270 cells, treatment with CDCA significantly increased *COX-2* mRNA expression in a time-dependent manner (CDCA, [0 h]; *P* = 1.0, [3 h]; *P* = 0.00000014, [6 h]; *P* = 0.00000014), but treatment with pH 4.5 medium had no effect on *COX-2* expression (pH 4.5, [0 h]; *P* = 1.0, [3 h]; *P* = 1.0, [6 h]; *P* = 0.98) (Fig. 4a). A similar phenomenon was observed in Het-1A cells (Fig. 4b). We then assessed the involvement of phospholipase A2 (PLA2), a synthetic enzyme of the cyclooxygenase substrate. The selective cytosolic PLA2 (cPLA2) inhibitor pyrrophenone inhibited PGE_2_ production induced by pH 4.5 medium (Pyrrophenone [0.2 μmol/L]; *P* = 0.98, Pyrrophenone [1 μmol/L]; *P* = 0.0029, Pyrrophenone [5 μmol/L]; *P* = 0.00065), but not by CDCA (Pyrrophenone [0.2 μmol/L]; *P* = 0.069, Pyrrophenone [1 μmol/L]; *P* = 0.99, Pyrrophenone [5 μmol/L]; *P* = 0.12), in KYSE-270 cells (Fig. 4c), suggesting the involvement of cPLA2 in pH 4.5-induced PGE_2_ production.

### Involvement of MAPKs in PGE_2_ production induced by pH 4.5 medium and CDCA

**Figure 5:**
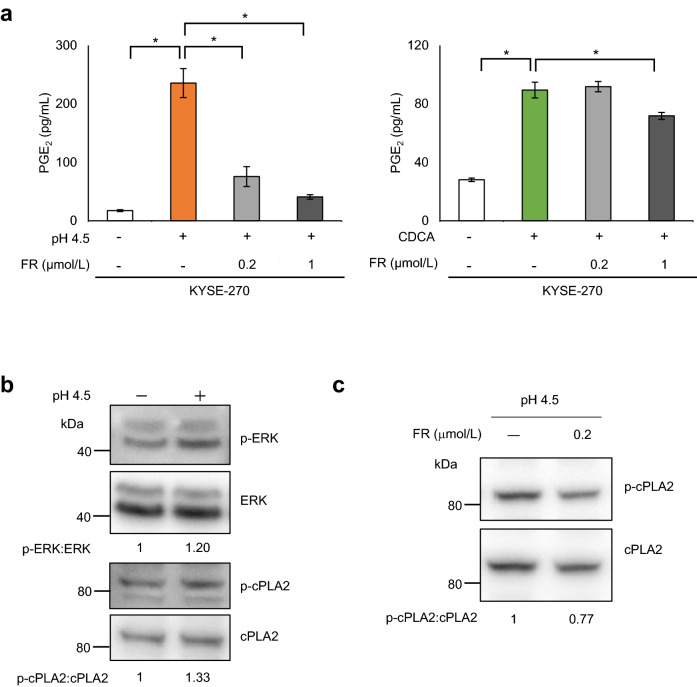
Different effects of pH 4.5 medium and CDCA on mitogen-activated protein kinase (MAPK) activation. (**a**) Treatment with the extracellular signal-regulated kinase (ERK) inhibitor FR180204 (FR; 0.2 and 1 μmol/L) inhibited PGE_2_ production induced by pH 4.5 medium but it had little or no effect on CDCA (400 μmol/L)-induced production in KYSE-270 cells. (**b**) ERK and cPLA2 phosphorylation were increased after pH 4.5 stimulation for 5 min. (**c**) pH 4.5-induced cPLA2 phosphorylation was inhibited by ERK inhibitor FR180204 (FR; 0.2 μmol/L) treatment. These shown blots were cropped to improve the conciseness and the full-length blots were presented in Supplementary Fig. S7. *PGE*_*2*_ prostaglandin E_2_; *CDCA* chenodeoxycholic acid; *cPLA2* cytosolic phospholipase A2. Data are presented as means ± SD (n = 3). Statistical significance was determined by Tukey–Kramer test; *P < .001.

Since MAPKs are involved in COX-2 expression and PLA2 activation^21–23^, we assessed their roles in PGE_2_ production to clarify the mechanisms underlying the observed effects of weak acids and bile acid. Among several MAPK inhibitors, we found that an ERK inhibitor (FR180204) prominently suppressed PGE_2_ production induced by pH 4.5 (FR180204 [0.2 μmol/L]; *P* = 0.00000020, FR180204 [01 μmol/L]; *P* = 0.00000020) but it had little effect on CDCA induced production (FR180204 [0.2 μmol/L]; *P* = 0.98, FR180204 [01 μmol/L]; *P* = 0.00044) (Fig. 5a). ERK phosphorylation level increase 5 min following treatment with pH 4.5 (Supplementary Fig. S2); simultaneously, cPLA2 phosphorylation levels were also increased (Fig. 5b). Moreover, ERK inhibitor (FR180204) suppressed cPLA2 phosphorylation under stimulation at pH 4.5 (Fig. 5c), indicating, at least in part, the involvement of ERK/cPLA2 in the PGE_2_ production by weak acid stimulation.

### Extracellular acid-sensing mechanism in oesophageal epithelial cells

**Figure 6:**
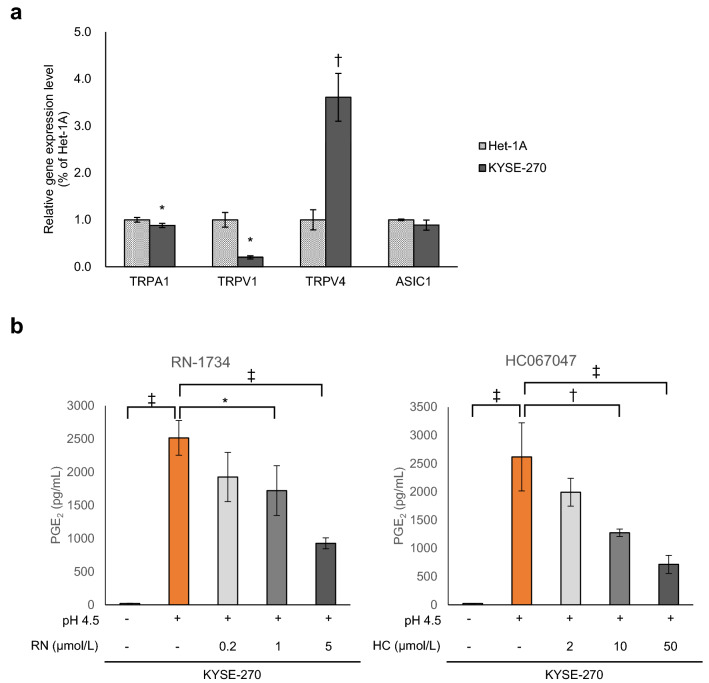
Involvement of transient receptor potential vanilloid 4 (TRPV4) in pH 4.5-induced PGE_2_ production in KYSE-270 cells. (**a**) Expression of *TRPV4* mRNA was higher in KYSE-270 cells than in Het-1A cells. (**b**) Treatment with the TRPV4 inhibitors RN-1734 (RN; 0.2, 1, 5 μmol/L) and HC067047 (HC; 2, 10, 50 μmol/L) significantly inhibited PGE_2_ production in KYSE-270 cells treated with pH 4.5 medium. *PGE*_*2*_ prostaglandin E_2_. Data are presented as means ± SD (n = 3). Statistical significance was determined by Student’s or Aspin–Welch's *t*-test or Tukey–Kramer test; *P < .05, ^†^*P* < .01, ^‡^*P* < .001.

In addition to the activation of ERK, an increase in intracellular calcium is essential for cPLA2 activation^24^. We confirmed that intracellular calcium increased in response to weak acid but not CDCA (Supplementary Fig. S3). We also investigated the expression levels of ion channels associated with acid-sensing and the regulation of calcium influx in normal cells and carcinoma cells. We found that TRPV4 expression was substantially higher in KYSE-270 cells than in Het-1A cells (*P* = 0.0012) (Fig. 6a). Moreover, two TRPV4 inhibitors, RN-1734 and HC067047, suppressed PGE_2_ production induced by pH 4.5 (RN-1734 [0.2 μmol/L]; *P* = 0.12, RN-1734 [1 μmol/L]; *P* = 0.028, RN-1734 [5 μmol/L]; *P* = 0.00019) (HC067047 [2 μmol/L]; *P* = 0.16, HC067047 [10 μmol/L]; *P* = 0.0020, HC067047 [50 μmol/L], *P* = 0.00012) (Fig. 6b). ERK phosphorylation was slightly increased by TRPV4 agonist treatment (Supplementary Fig. S4), indicating the involvement of TRPV4/ERK/cPLA2 pathway in the weak acid-induced PGE_2_ production, at least in part.

On the contrary, TRPV1 expression was lower in KYSE-270 cells than in Het-1A cells (Fig. 6a), while TRPV1 antagonist did not suppress PGE_2_ production induced by weak acid stimulation in KYSE-270 cells (Supplementary Fig. S5).

### Effect of HST on PGE_2_ production by pH 4.5 medium and CDCA

**Figure 7:**
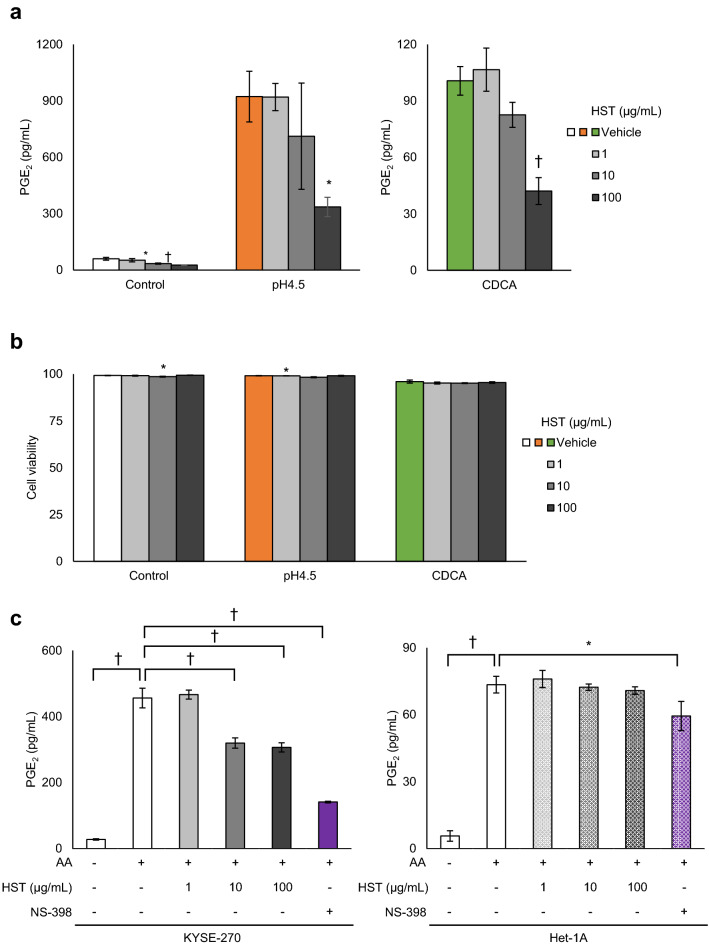
Effects of Hangeshashinto (HST) on PGE_2_ production and viability in oesophageal epithelial cells. (**a**) Treatment with HST (1, 10, and 100 μg/mL) significantly inhibited PGE_2_ production in KYSE-270 cells treated with pH 4.5 medium or with 400 μmol/L CDCA. (**b**) HST (1, 10, and 100 μg/mL) had no effect on viability in KYSE-270 cells. (**c**) HST (1, 10, and 100 μg/mL) and NS-398 (0.2 μmol/L) inhibited PGE_2_ production induced by arachidonic acid (AA) supplementation (3 μmol/L, 15 min) in KYSE-270 cells but not in Het-1A cells. *PGE*_*2*_ prostaglandin E_2_; *CDCA* chenodeoxycholic acid. Data are presented as means ± SD (n = 3). Statistical significance was determined by Dunnett's or Tukey–Kramer test; *P < .01, ^†^*P* < .001.

In KYSE-270 cells, HST treatment inhibited both pH 4.5- and CDCA- induced PGE_2_ production (pH 4.5; HST [1 μg/mL]; *P* = 0.74, HST [10 μg/mL]; *P* = 0.17, HST [100 μg/mL]; *P* = 0.0029) (CDCA; HST [1 μg/mL]; *P* = 0.73, HST [10 μg/mL]; *P* = 0.07, HST [100 μg/mL]; *P* = 0.0000077) (Fig. 7a) but did not affect cell viability (Fig. 7b). To assess the effect of HST on PGE_2_ synthesis, we measured the PGE_2_ production induced by AA. In KYSE-270 cells, the increase in PGE_2_ production by the addition of AA was suppressed by HST in a dose-dependent manner (HST [1 μg/mL]; *P* = 0.96, HST [10 μg/mL]; *P* = 0.00000048, HST [100 μg/mL]; *P* = 0.00000033) and was also suppressed by a COX-2 inhibitor (NS-398) (Fig. 7c). AA-induced PGE_2_ production was minimally affected by HST (HST [1 μg/mL]; *P* = 0.95, HST [10 μg/mL]; *P* = 1.0, HST [100 μg/mL]; *P* = 0.95) and NS-398 in Het-1A cells (Fig. 7c).

## Discussion

**Figure 8:**
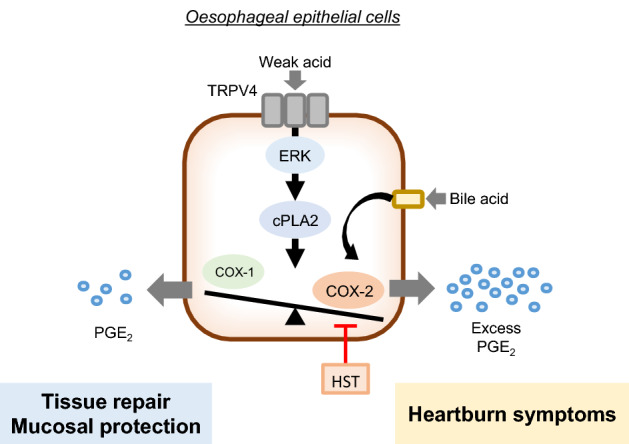
A graphical hypothesis of this study. Weak acid exposure at pH 4–5 induces PGE_2_ production via TRPV4/ERK/cPLA2. Under normal conditions, oesophageal epithelial cells produce only a small amount of PGE_2_ in response to weak acid stimulation, which may contribute to biological protection. Meanwhile, in cases of high expression of COX-2 due to stimulations, such as CDCA, weak acid exposure at pH 4–5 produces excess PGE_2_, which may trigger various symptoms including heartburn and oesophageal cancer development. HST is effective in treating various GERD symptoms due to excessive PGE_2_ production by suppressing COX-2-dependent production. *PGE*_*2*_ prostaglandin E_2_; *TRPV4* transient receptor potential vanilloid 4; *ERK* extracellular signal-regulated kinase; *cPLA2* cytosolic phospholipase A2; *COX-2* cyclooxygenase-2; *CDCA* chenodeoxycholic acid; *HST* hangeshashinto.

The development of an excellent surgical animal model for GERD has contributed to the elucidation of the pathophysiological mechanisms underlying GERD^25^, but many unresolved issues remain. In particular, there has been little progress in research on the effect of reflux materials with a range of pH values on GERD symptoms owing to the limitations of animal models for such detailed investigations. In the present study, we demonstrated for the first time that PGE_2_ production increases in oesophageal epithelial cells in response to a narrow pH range of pH 4–5 via TRPV4/ERK/cPLA2 (Fig. 8). These results indicate that weak acids (i.e. pH 4–5) could contribute to GERD symptoms like heartburn via PGE_2_ production.

PGE_2_ is involved in the induction of heartburn symptoms^14,26^. Interestingly, heartburn symptoms were most frequently reported when weak acid was refluxed, especially at pH 5, in patients with PPI-refractory GERD^10,11^. Moreover, the administration of weak acids (pH 4–5) induced heartburn symptoms in nearly 50% of patients with GERD symptoms^27^. Although increase in PGE_2_ production by oesophagus acid exposure is reported in healthy volunteers, the relation between PGE_2_ production and precise extracellular pH has not been fully investigated^15^. In the present study, we demonstrated that weak acids, at pH 4–5, significantly induced the production of PGE_2_ in human oesophageal squamous epithelial cell carcinoma (KYSE-270). Similar results were obtained in normal oesophageal epithelial squamous cells (Het-1A) and normal rat oesophageal mucosa (in vivo), suggesting a widely applicable phenomenon in oesophagus epithelial cells. Our results indicate that excessive PGE_2_ production by oesophageal epithelial cells induced by weak acids (pH 4–5) may explain heartburn symptoms observed in patients with PPI-refractory GERD. Moreover, we found that PGE_2_ production increased as pH decreased from 4.7, peaked at pH 4.4, and gradually reduced thereafter due to increased cytotoxicity in KYSE-270 cells. Until now, acid reflux in the oesophagus with pH values < 4 has been a focus of GERD diagnosis, and reducing the reflux time with pH < 4 has been considered important in PPI therapy^28^. However, our data suggest that careful attention should be paid not only to acid reflux with pH < 4 but also to weak acid reflux with pH 4–5. PGE_2_ is suggested to be involved in the exacerbation of various gastrointestinal cancers, including oesophageal cancer^29,30^. However, there are no reports regarding the possible involvement of weak acid reflux in oesophageal cancer. In this study, weak acid stimulation significantly induced PGE_2_ production in human oesophageal squamous epithelial cell carcinoma (KYSE-270) but not oesophagus adenocarcinoma cells (FLO-1 and KYAE-1). Although weak acid reflux may play a role in exacerbating oesophageal cancer through PGE_2_ production in the oesophageal mucosa, further in vivo investigations are required to verify the involvement of weak acids in oesophageal cancer.

In this study, we showed that PGE_2_ production in response to pH 4.5 is mediated by cPLA2 activation, since its inhibitor suppressed PGE_2_ production induced by a weak acid in KYSE-270 cells. ERK activation and calcium influx are considered important for cPLA2 activation^24,31^; thus, we also confirmed that intracellular calcium levels were increased by weak acidification of the medium, and weak acid-induced cPLA2 phosphorylation was suppressed by ERK inhibitor treatment. Furthermore, ERK phosphorylation was increased by TRPV4 agonist treatment. Our data indicated that TRPV4/ERK/cPLA2 are involved in weak acid-induced PGE_2_ production. Recently, not only acid reflux but also bile acid reflux has been attracting attention in GERD pathogenesis^32^. Interestingly, bile acid-induced PGE_2_ production was not affected by cPLA2 and ERK inhibitors despite the ERK activation, and bile acid stimulation had no effect on calcium influx. Bile acids strongly induce COX-2 expression and PGE_2_ production in oesophageal epithelial cells^18^, which is consistent with our results in KYSE-270 cells. In Het-1A cells, PGE_2_ production was not elevated by CDCA stimulation, although *COX-2* expression was significantly increased. This might be attributed to the significantly lower basal and induced expression levels of *COX-2* compared to those in KYSE-270 cells. Interestingly, pH 4.5 stimulation induced PGE_2_ production without the induction of *COX-2* expression in both Het-1A cells and KYSE-270 cells, indicating that the mechanisms underlying PGE_2_ production differ between weak acid and bile acid stimulation.

Previous studies have shown that TRPV4 is involved in the regulation of intracellular calcium in oesophagus epithelial cells, although its physiological role remains unclear^33,34^. TRPV4 is reportedly activated by changes in not only temperature and osmotic pressure but also extracellular pH^35^. Particularly, TRPV4 begins to be activated below pH 6 and most potent activation were observed at around pH 4^36^. In the present study, we showed that TRPV4 is more highly expressed in oesophageal epithelial squamous cell carcinoma cells (KYSE-270) than in normal oesophageal cells. Two kinds of TRPV4 inhibitors significantly suppressed PGE_2_ production induced by pH 4.5. We also confirmed that TRPV4 agonist increased ERK phosphorylation, which is essential for cPLA2 activation, suggesting a novel physiological role of TRPV4 in oesophageal epithelial cells. TRPV1, which may be a crucial factor involved in oesophageal hyperesthesia, plays a role in pH sensing of weak acid^37,38^. However, TRPV1 inhibitor did not suppress weak acid-induced PGE_2_ production in our study. The suppressive effect of the TRPV4 inhibitor on PGE_2_ production was limited and further investigations of other acid-sensing mechanisms are warranted.

HST, a Japanese traditional medicine (Kampo medicine), contains the extracts of seven medicinal herbs and has been approved by Japanese Ministry of Health, Labour and Welfare for clinical use^39^. HST is used for the treatment of inflammatory diarrhoea, gastritis, and heartburn and is effective for the treatment of stomatitis and diarrhoea via the reduction of PGE_2_ production^40–44^. Combined treatment with PPI and HST is effective for alleviating heartburn symptoms in patients with PPI-refractory GERD to the same extent as a double dose of PPI^19^. Furthermore, HST significantly inhibited carcinogenesis in a surgical rat reflux model^45^. In this study, we showed that HST suppressed both weak acid- and bile acid-induced PGE_2_ production in oesophagus epithelial cells. These findings suggest that oesophageal PGE_2_ suppression could relieve the clinical symptoms of PPI-refractory GERD in patients exhibiting weak acid reflux. HST suppresses PGE_2_ production by inhibiting COX-2 activity, not COX-1 activity, as confirmed using recombinant proteins^46^. Moreover, components of HST, especially ginger-derived, are reported to suppress COX-2 activity in several studies^42,47,48^. Additionally, we showed that HST prevented AA-induced PGE_2_ production only in KYSE-270 cells, but not in Het-1A cells with low COX-2 expression. The suppressive effect of the COX-2 inhibitor NS-398 on PGE_2_ production was stronger in KYSE-270 cells than in Het-1A cells, suggesting that HST supressed COX-2-dependent PGE_2_ production. Excessive PGE_2_ production is involved in pain and tumour progression, while a small amount of PGE_2_ derived from COX-1 is important in tissue repair and gastrointestinal mucosal protection^30,49^. Thus, the selective effect of HST on COX-2 may contribute to the alleviation of GERD without disrupting mucosal protection. In the future, we hope to further examine the safety of HST and to clarify the efficacy of HST in patients with GERD in greater detail.

In summary, we demonstrated that weak acid reflux induced PGE_2_ production by oesophagus epithelial cells via a unique mechanism, which may be involved in the pathogenesis of refractory GERD. Therefore, pH correction up to 5 in patients with GERD may prevent the heartburn symptoms.

## Methods

### Reagents

HST was obtained by spray-drying a hot water extract of a mixture of seven crude drugs: Pinellia tuber (*Pinelliae tuber*) 5.0 g, Scutellaria root (*Scutellariae radix*) 2.5 g, Glycyrrhiza (*Glycyrrhizae radix*) 2.5 g, Jujube (*Zizyphi fructus*) 2.5 g, Ginseng (*Ginseng radix*) 2.5 g, Processed ginger (*Zingiberis processum rhizome*) 2.5 g, and Coptis rhizome (*Coptidis rhizome*) 1.0 g. HST was suspended in phosphate-buffered saline (PBS) at 100 mg/mL and added to the culture medium at final concentrations of 1–100 µg/mL.

### Cell culture

Human oesophageal squamous cell carcinoma KYSE-270 cells (ECACC, Salisbury, UK, RRID:CVCL 1350) were cultured in F12/RPMI-1640 medium (1:1) supplemented with 100 units/mL penicillin G and 0.1 mg/mL streptomycin containing 2% foetal bovine serum and 2 mmol/L glutamine at 37 °C and 5% CO_2_. Human normal oesophageal epithelial cells Het-1A (ATCC, Manassas, VA, USA, RRID: CVCL_3702) were cultured in Bronchial Epithelial Cell Growth Medium (BEGM) supplemented with 100 units/mL penicillin G and 0.1 mg/mL streptomycin at 37 °C and 5% CO_2_. Human oesophageal adenocarcinoma KYAE-1 cells (JCRB cell bank, Osaka, Japan, RRID: CVCL_1825) were cultured in F12/RPMI-1640 medium (1:1) supplemented with 100 units/mL penicillin G and 0.1 mg/mL streptomycin containing 5% foetal bovine serum and 2 mmol/L glutamine at 37 °C and 5% CO_2_. Human oesophageal adenocarcinoma FLO-1 cells (ECACC, Salisbury, UK, RRID: CVCL_2045) were cultured in DMEM supplemented with 100 units/mL penicillin G and 0.1 mg/mL streptomycin containing 10% foetal bovine serum and 2 mmol/L glutamine at 37 °C and 5% CO_2_.

### Cell treatment

Cells were seeded in 96-well plates (Het-1A cells; 5.0 × 10^4^/well, KYSE-270 cells; 2.5 × 10^4^/well) or 24-well plate (Het-1A cells; 5.0 × 10^5^/well, KYSE-270 cells; 3.0 × 10^5^/well) or 12-well plates (KYSE-270 cells; 5.0 × 10^6^/well) and incubated overnight. The cells were treated for 2 h in acidic culture conditions (pH 6.5 to pH 3.5) or with chenodeoxycholic acid (CDCA; 200 or 400 μmol/L) (Wako Chemical, Osaka, Japan). The cells were then washed and cultured in fresh pH 7.2 medium for an additional 6 h. HST (1, 10, and 100 μg/mL) or inhibitors [NS-398 (Wako Chemical), pyrrophenone (Merck & Co., Kenilworth, NJ, USA), FR180204 (Merck & Co.), RN-1734 (Wako Chemical), HC067047 (Wako Chemical), AMG9810 (Tocris Bioscience, Bristol, UK), A784168 (Tocris Bioscience), GSK1016790A (Sigma-Aldrich, St. Louis, MO, USA)] were added to both acidic and pH 7.2 culture medium for 8 h. Finally, the culture medium was collected to estimate PGE_2_ concentrations using the PGE_2_ Enzyme Immunoassay Kit (Cayman Chemical Co., Ann Arbor, MI, USA).

### Measurement of weak acid-induced PGE_2_ production in rats (in vivo)

Animal experimental procedures were performed according to the Guidelines for the Care and Use of Laboratory Animals and approved by the Laboratory Animal Committee (permit no. 20–018) of Tsumura & Co. (Tokyo, Japan).

Six-week-old male Sprague–Dawley rats weighing 180–200 g were purchased from Charles River Laboratories Japan, Inc. (Kanagawa, Japan). All animals were housed in a room with controlled ambient temperature (23 ± 3 °C), humidity (50 ± 20%), and lighting (12 h light–dark cycle) conditions. The animals were provided with ad libitum water and a standard laboratory animal diet (MF; Oriental Yeast Co., Ltd., Tokyo, Japan).

Rats (n = 10) were anesthetized with urethane (Sigma-Aldrich) and α-chloralose (Wako Chemical) and injected with the analgesic agent Vetorphale (Meiji Seika Pharma, Tokyo, Japan). The oesophagus was orally cannulated with airway management, after which warmed weak acid solution (pH 4.5) or PBS was perfused at approximately 500 μL/min for 1 h using a perfusion pump. The perfusate was drained outside from the cannula inserted into the oesophagus and emerged from the stomach and collected for the last 5 min. The samples were concentrated using a Sep-Pak C18 cartridge (Waters, Milford, MA, USA) to assess PGE_2_ concentration.

To measure the capacity of PGE_2_ production by the rat oesophageal mucosa, rats were euthanized after perfusion and the oesophageal mucosa was collected. The mucosa was divided into proximal, intermediate, and distal regions and incubated in F12/RPMI-1640 medium (1:1) for 2 h at 37 °C. PGE_2_ concentration in the medium was then measured; PGE_2_ production capacity was calculated by dividing the total amount of PGE_2_ in the medium by the amount of total protein in each mucosal tissue. The data were shown as the average values of the three regions (proximal, intermediate, and distal) per hour.

### Measurement of PGE_2_ synthetic capacity using intact cells

Enzymatic activity related to PGE_2_ synthesis was determined by measuring the accumulation of PGE_2_ induced by arachidonic acid (AA; Wako Chemical) in the culture fluids. Briefly, Het-1A cells (5.0 × 10^4^/well) and KYSE-270 cells (2.5 × 10^4^/well) were cultured overnight in 96-well plates. The culture fluids were replaced with the same fresh medium containing HST or the COX-2 inhibitor NS-398 for 15 min, and AA was added to the culture medium at a final concentration of 3 μmol/L. PGE_2_ concentrations were measured as described above after further incubation for 15 min.

### Gene expression analysis

To measure mRNA expression, real-time qRT-PCR with TaqMan technology (Applied Biosystems, Warrington, UK) was used. Cells were lysed in QIAzol Lysis Reagent (Qiagen, Valencia, CA, USA), and total RNA was isolated using an RNeasy Kit (Qiagen) according to the manufacturer’s instructions. The cDNA was prepared using a High-capacity RT Kit (Applied Biosystems). PCR was performed using the ABI Prism 7900 sequence detector (Applied Biosystems) with default parameters. Sample-to sample variation in RNA loading was controlled by comparison with *ACTB* or *GAPDH*. The primer/probe sets were as follows: *COX-1* (Hs00377726_m1), *COX-2* (Hs00153133_m1), *TRPA1* (Hs00175798_m1), *TRPV1* (Hs00218912_m1), *TRPV4* (Hs01099348_m1), *ASIC1* (Hs00952807_m1), *ACTB* (Hs01060665_g1), and *GAPDH* (Hs02786624_g1).

### Cytotoxicity assay

Cytotoxicity was assayed using the LDH-Cytotoxic Test Kit (Wako Chemical). The accurate estimation of LDH activity in acidic conditions is challenging; accordingly, cytotoxicity was evaluated by examining the amount of LDH remaining in living cells. After stimulation, the cells were solubilised by cell lysis buffer (Cell Signaling Technology, Danvers, MA, USA) containing 1% Triton X-100, and the supernatant was used for the LDH assay after centrifugation. Cytotoxicity was calculated using the following formula for relative LDH activity: Cell death (%) = 100 × [(b − a)/b], where a = absorbance at 560 nm for the test sample, b = control.

Cell viability after HST treatment was evaluated by the amount of LDH released in the medium. As a positive control, cells were treated with 0.2% Tween 20 for 15 min and the medium was collected. Cell viability was calculated using the following formula for relative LDH activity: viability (%) = 100 × [(c − a)/(c − b)], where a = test sample, b = blank well, and c = positive control. Absorbance was measured using a microplate reader SpectraMax Plus 384 (Molecular Devices, San Jose, CA, USA).

### Cell metabolic activity assay

Cell metabolic activity was evaluated using a Cell Counting Kit-8 (CCK-8; Dojindo Laboratories, Kumamoto, Japan). After stimulation, 8 µL of CCK-8 reagent was added to 100 µL of culture medium, and the plates were incubated at 37 °C in an atmosphere of 5% CO_2_ for 1 h. Cell metabolic activity is presented as the change in absorbance at 450 nm, as determined using a microplate reader (SpectraMax Plus 384).

### Immunoblotting analysis

Cells were lysed with a cell lysis buffer (Cell Signaling Technology) containing 1% Triton X-100, Protease Inhibitor Cocktail (Sigma-Aldrich), and Phosphatase Inhibitor Cocktail 3 (Sigma-Aldrich). After centrifugation at 10,000×*g* for 15 min, the supernatants were collected. The cell lysates were then fractionated by SDS–polyacrylamide gel electrophoresis and electroblotted onto PVDF membranes. The membranes were probed with primary antibodies and HRP-conjugated secondary antibodies. Protein was detected using the ECL system and analysed using a ChemiDoc system (Bio-Rad Laboratories, Hercules, CA, USA). The following primary antibodies were used: anti-phospho-ERK (Thr202/tyr204; #4377, RRID: AB_331775), anti-ERK (#4695, RRID: AB_390779), anti-COX2 (#4842, RRID: AB_2084968), anti-cPLA2 (#2832S, RRID: AB_2164442), anti-phospoh-cPLA2 (#2831S, RRID: AB_2164445) and anti-GAPDH (#2118, RRID: AB_561053), all from Cell Signaling Technology. HRP-conjugated anti-rabbit IgG (NA934; GE Healthcare, Chicago, IL, USA) was used as the secondary antibody.

### Ca^2+^ measurements

KYSE-270 cells were loaded with 5 μM Fura-2-AM (Dojindo Laboratories) in HBSS buffer for 60 min at 37 °C. Fura-2 fluorescence intensity was measured using a fluorescence microplate reader (FlexStation 3, Molecular Devices, ex: 340 or 380 nm, em: 510 nm). Intracellular Ca^2+^ concentration was evaluated as the change in ratio of fluorescence intensity exited at 340 to that at 380 nm.

### Statistical analyses

Data are reported as means ± standard deviation. Student’s or Aspin-Welch’s t-test was performed for two-group comparisons, and the Tukey–Kramer or Dunnett test for multiple-group comparisons. Statistical differences were analysed using StatLight (Yukms Co. Ltd., Kawasaki, Japan). *P* < 0.05 was considered statistically significant.

## Supplementary information

Supplementary Information.

## Data Availability

All data generated or analysed during this study are included in this published article.
