# Supplementary material for: Weak acids induce PGE2 production in human oesophageal cells: novel mechanisms underlying GERD symptoms
Source: Sci Rep. 2020 Nov 27;10:20775. doi: 10.1038/s41598-020-77495-z (PMC7695745; doi:10.1038/s41598-020-77495-z)
Supplement: Supplementary file 1 — Supplementary Information. [file 41598_2020_77495_MOESM1_ESM.pdf]

Title : Weak acids induce PGE_2 production in human oesophageal cells: novel mechanisms underlying GERD symptoms

Authors: Daichi Sadatomi[1], Toru Kono[2*], Sachiko Mogami[1], and Naoki Fujitsuka[1].

1. Tsumura Kampo Research Laboratories, Tsumura & Co., Ibaraki, Japan.
2. Institute of Biomedical Research, Sapporo Higashi Tokushukai Hospital, Hokkaido, Japan.

***Correspondence:** Toru Kono, M.D., Ph.D., F.A.C.S.
Institute of Biomedical Research, Sapporo Higashi Tokushukai Hospital
3-1, N-33, E-14, Higashi-ku, Sapporo, Hokkaido 065-0033, Japan
Phone: +81-11 722 1110 (ext. 2736)
Fax: +81-11 788 3628
Email addresses: kono@toru-kono.com

Supplemental Figure S1

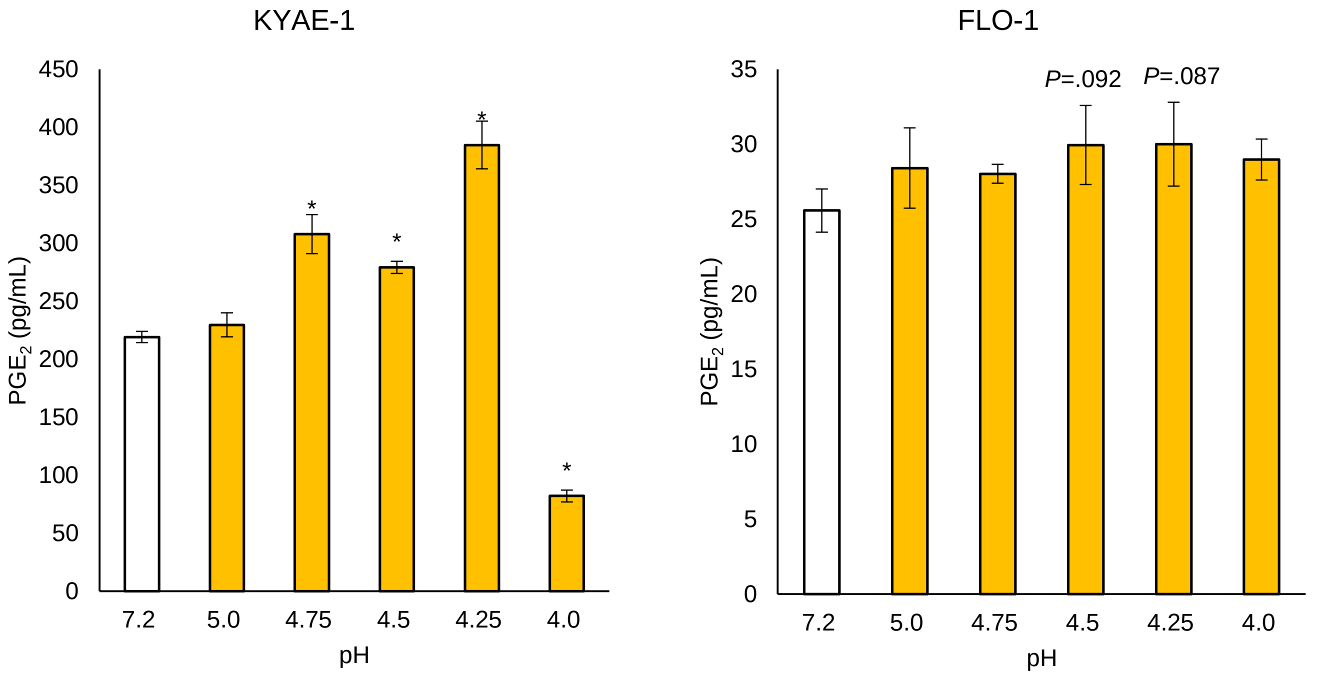

Supplemental Figure S1. PGE$_2$ production in oesophageal adenocarcinoma cells. In oesophageal adenocarcinoma KYAE-1 cells, PGE$_2$ production was slightly increased upon culture in fresh medium with pH 7.2 for 6 h after exposure to pH 4.25 to 4.75 medium for 2 h. Conversely, PGE2 production was not significantly increased in FLO-1 cells under the same conditions. Data are presented as means ± SD (n=3). Statistical significance was determined by Dunnett's test; $^*P < .001$, compared with pH 7.2 medium.

Supplemental Figure S2

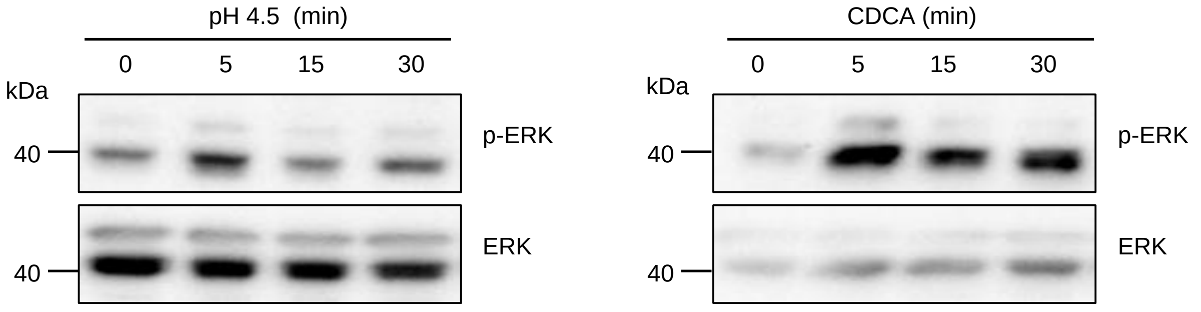

Supplemental Figure S2. pH 4.5 and CDCA induced ERK phosphorylation. ERK phosphorylation was increased after pH 4.5 stimulation but was persistently increased by CDCA stimulation, as shown by western blot analysis. The shown blots were cropped to improve the conciseness, and the full-length blots are presented in Supplementary Fig. 8.

Supplemental Figure S3

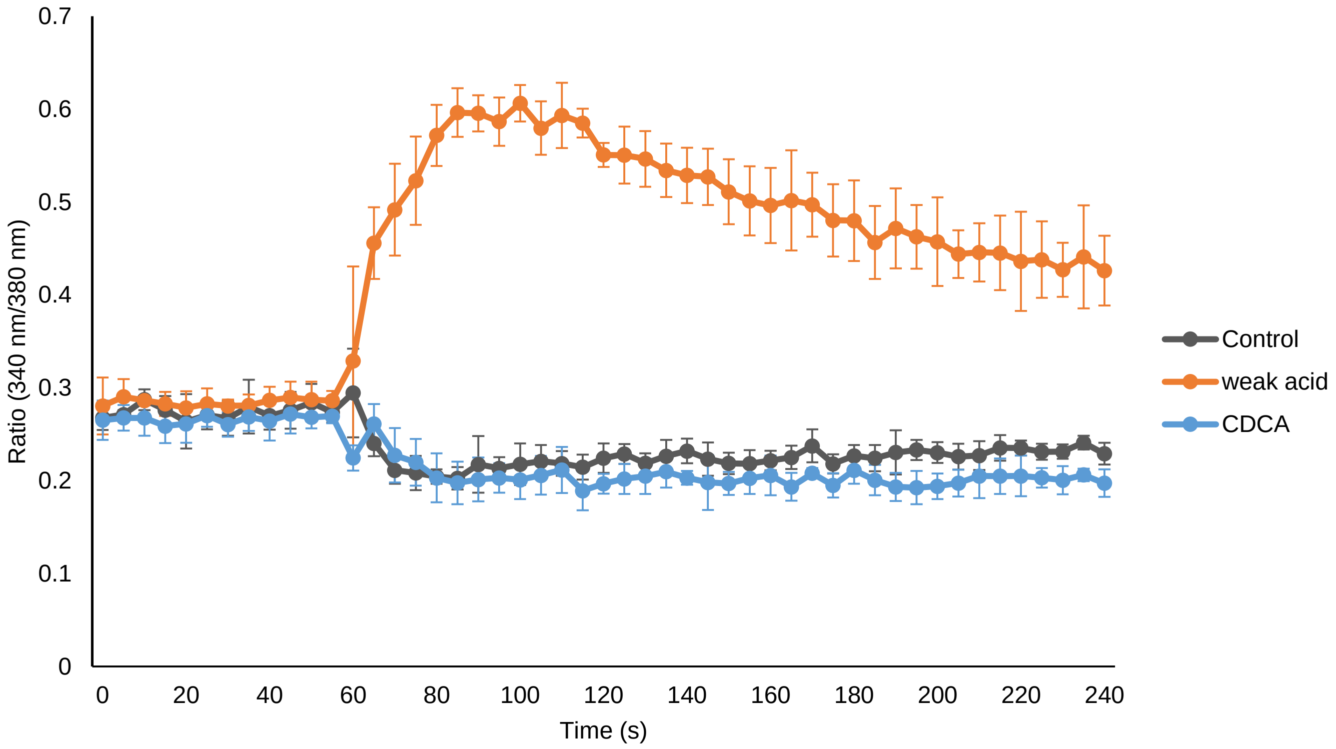

Supplemental Figure S3. Weak acid induced elevation of intracellular calcium. Fura-2 AM, a calcium indicator, showed that intracellular calcium was increased by pH 4.5 stimulation, but not by CDCA, in KYSE-270. Data are presented as means ± SD (n=6).

Supplemental Figure S4

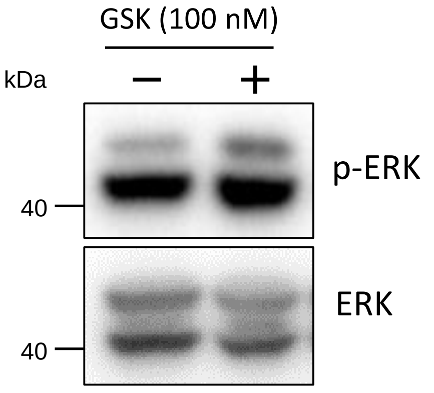

Supplemental Figure S4. Relationship between ERK and TRPV4.
In KYSE-270 cells, TRPV4 agonist GSK-1016790A (100 nM, 5 min) induced ERK phosphorylation. The shown blots were cropped to improve the conciseness, and the full-length blots are presented in Supplementary Fig. 9.

Supplemental Figure S5

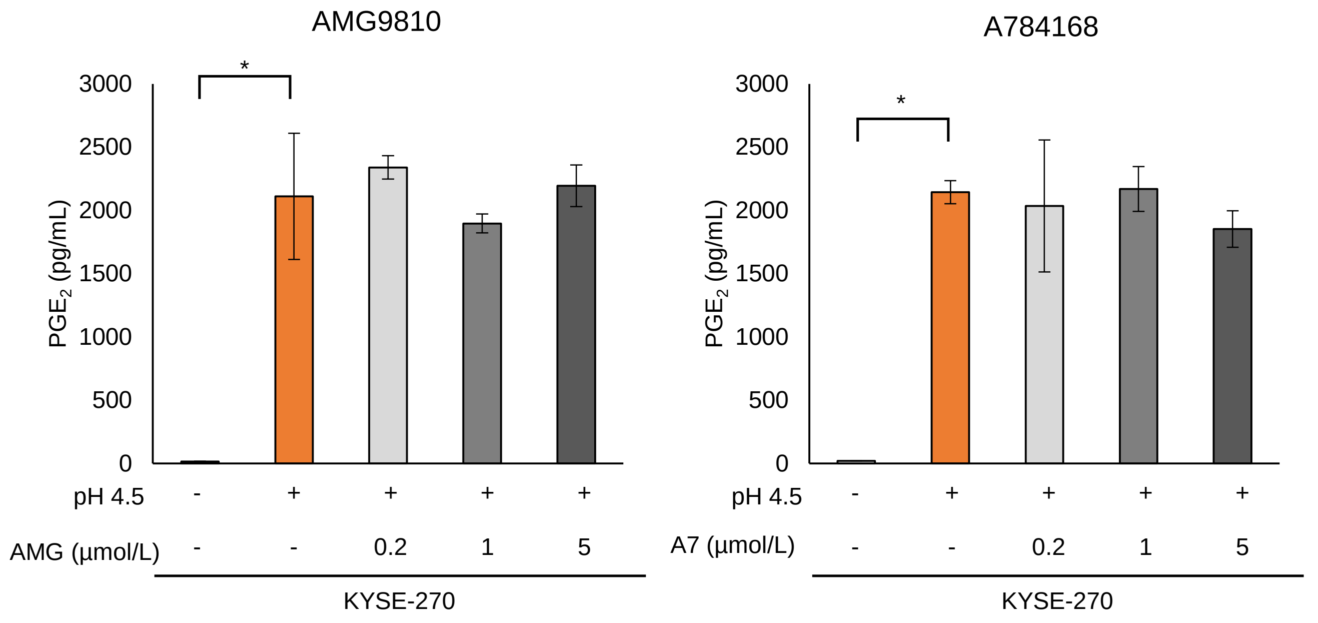

Supplemental Figure S5. TRPV1 is not involved in pH 4.5-induced PGE_2 production in KYSE-270 cells.

Treatment with the TRPV1 inhibitors AMG9810 (AMG; 0.2, 1 and 5 µmol/L) and A784168 (A7; 0.2, 1 and 5 µmol/L) did not inhibit PGE_2 production in KYSE-270 cells treated with pH 4.5 medium. Data are presented as means ± SD (n = 3). Statistical significance was determined by Tukey-Kramer test; *$P < .001$.

Supplemental Figure S6

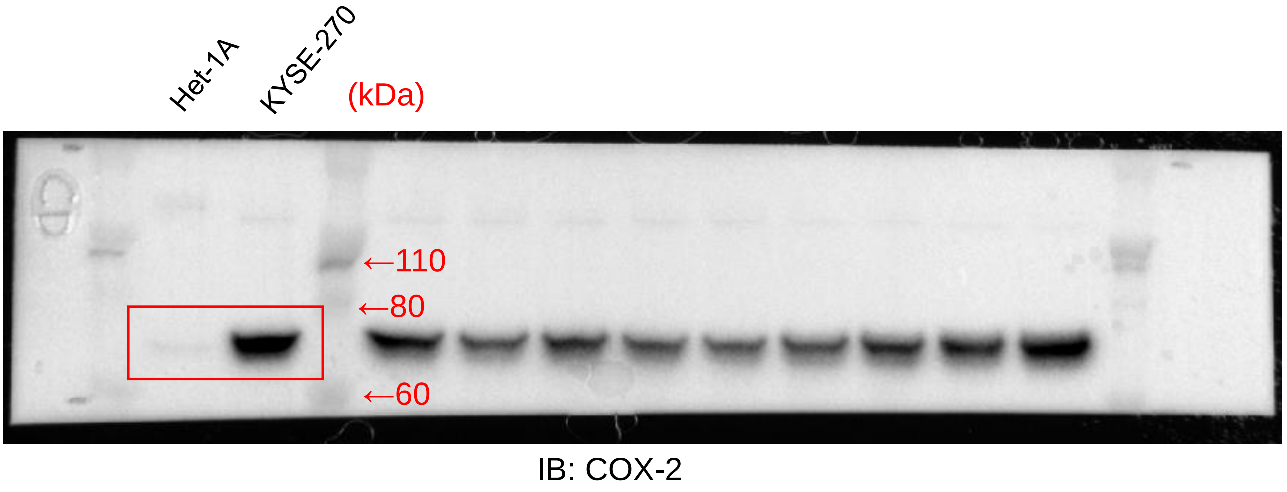

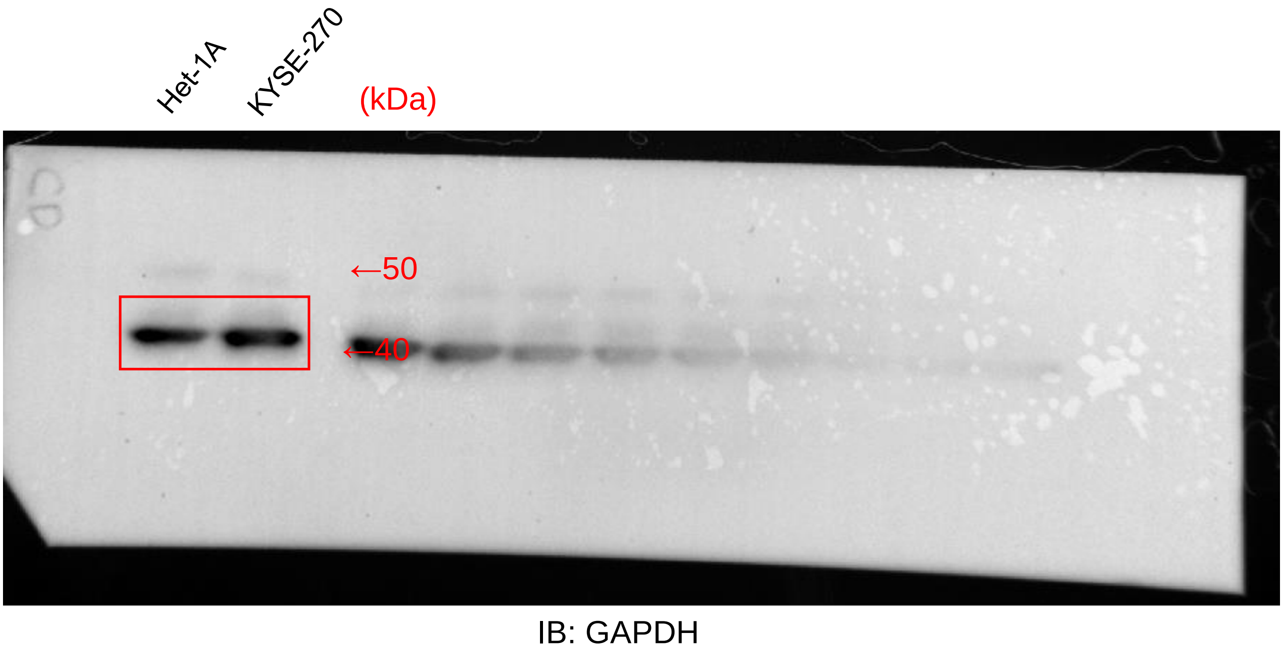

Supplementary Figure S6. Full-length blots of Fig. 3b.

Supplemental Figure S7

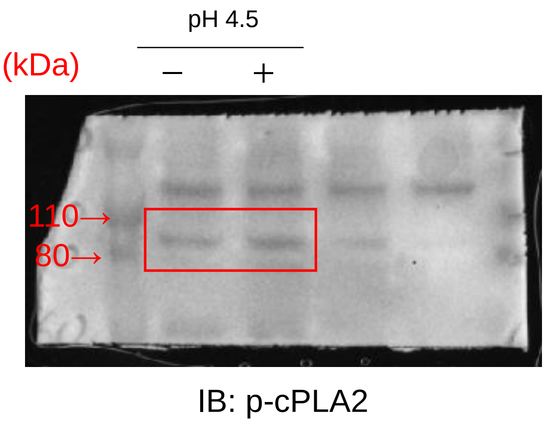

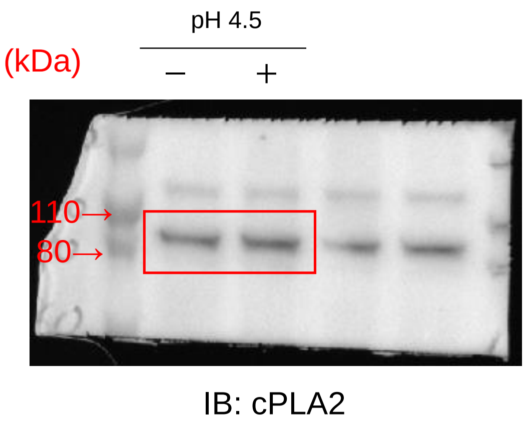

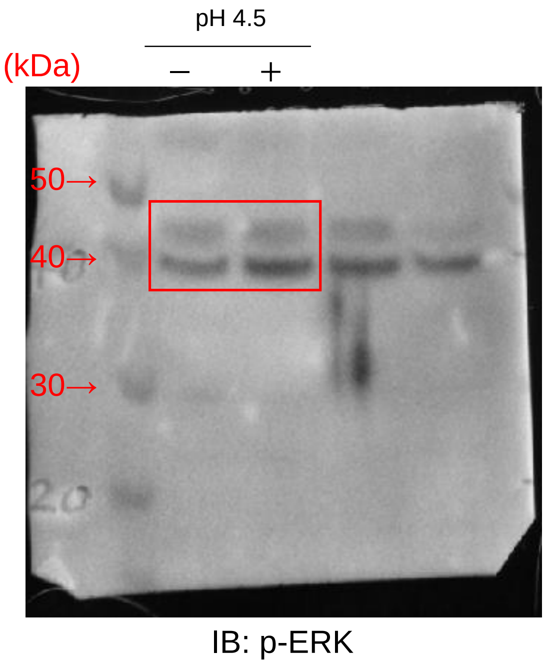

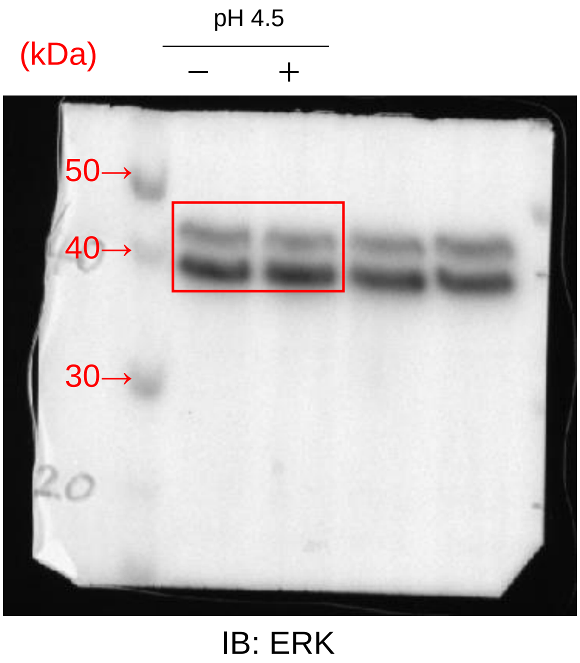

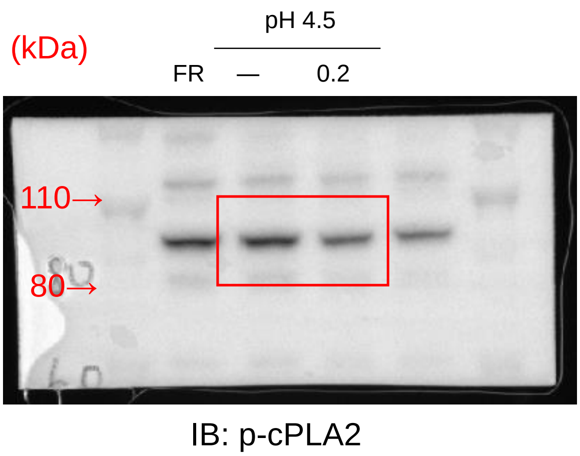

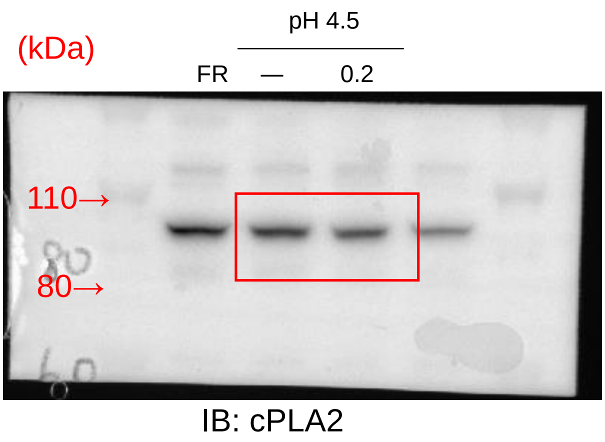

Supplementary Figure S7. Full-length blots of Fig. 4b and 4c.

Supplemental Figure S8

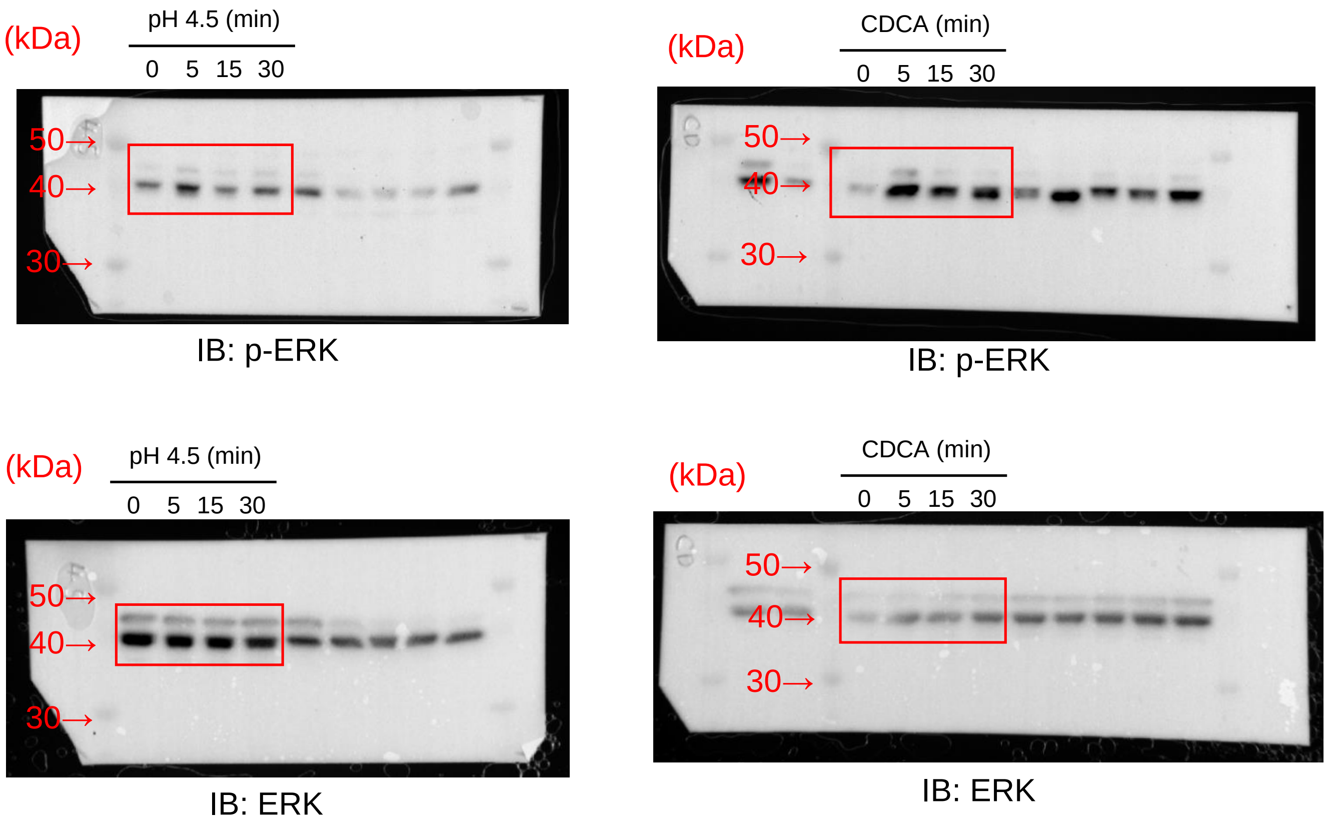

Supplementary Figure S8. Full-length blots of Supplemental Fig. S2.

Supplemental Figure S9

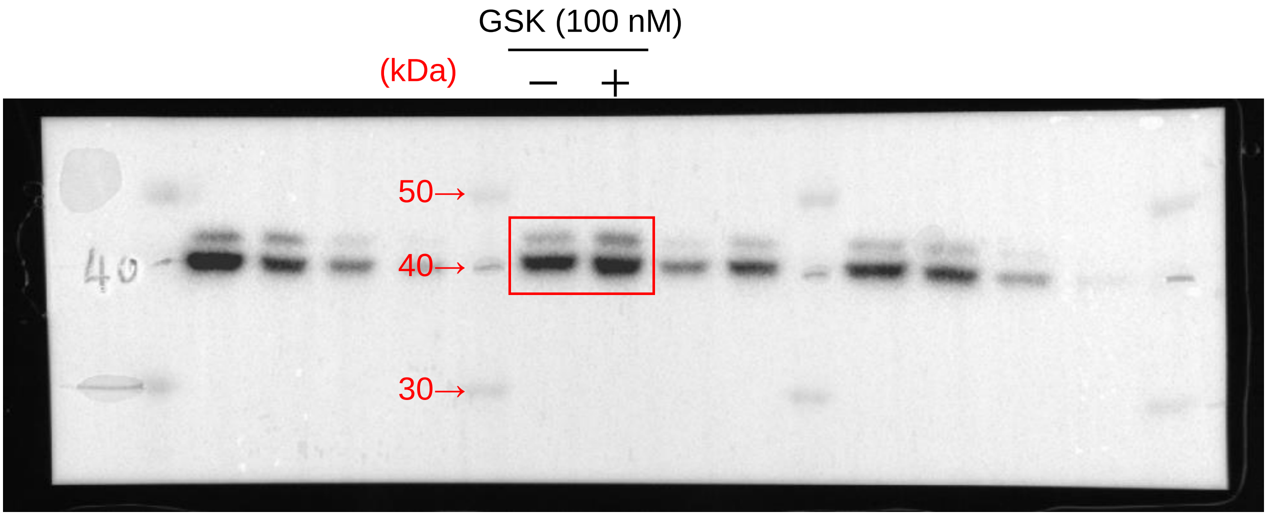

IB: p-ERK

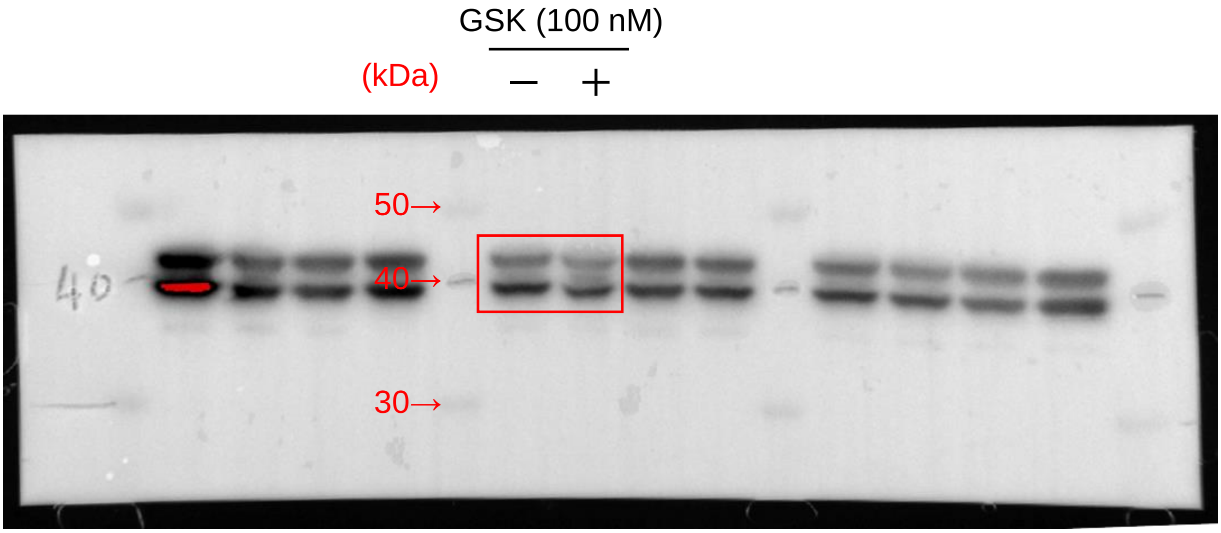

IB: ERK

Supplementary Figure S9. Full-length blots of Supplemental Fig. S4.
